# New Insights into Somatic Embryogenesis: *LEAFY COTYLEDON1, BABY BOOM1* and *WUSCHEL-RELATED HOMEOBOX4* Are Epigenetically Regulated in *Coffea canephora*

**DOI:** 10.1371/journal.pone.0072160

**Published:** 2013-08-20

**Authors:** Geovanny I. Nic-Can, Adolfo López-Torres, Felipe Barredo-Pool, Kazimierz Wrobel, Víctor M. Loyola-Vargas, Rafael Rojas-Herrera, Clelia De-la-Peña

**Affiliations:** 1 Campus de Ciencias Exactas e Ingeniería, Universidad Autónoma de Yucatán, Mérida, Yucatán, México; 2 Centro de Investigación Científica de Yucatán, Unidad de Bioquímica y Biología Molecular de Plantas, Mérida, Yucatán, México; 3 Facultad de Química, Universidad de Guanajuato, Guanajuato, México; 4 Unidad de Biotecnología, Centro de Investigación Científica de Yucatán, Mérida, Yucatán, México; Universidad Miguel Hernández de Elche, Spain

## Abstract

Plant cells have the capacity to generate a new plant without egg fertilization by a process known as somatic embryogenesis (SE), in which differentiated somatic cells can form somatic embryos able to generate a functional plant. Although there have been advances in understanding the genetic basis of SE, the epigenetic mechanism that regulates this process is still unknown. Here, we show that the embryogenic development of *Coffea canephora* proceeds through a crosstalk between DNA methylation and histone modifications during the earliest embryogenic stages of SE. We found that low levels of DNA methylation, histone H3 lysine 9 dimethylation (H3K9me2) and H3K27me3 change according to embryo development. Moreover, the expression of *LEAFY COTYLEDON1* (*LEC1*) and *BABY BOOM1* (*BBM1)* are only observed after SE induction, whereas *WUSCHEL-RELATED HOMEOBOX4* (*WOX4*) decreases its expression during embryo maturation. Using a pharmacological approach, it was found that 5-Azacytidine strongly inhibits the embryogenic response by decreasing both DNA methylation and gene expression of *LEC1* and *BBM1*. Therefore, in order to know whether these genes were epigenetically regulated, we used Chromatin Immunoprecipitation (ChIP) assays. It was found that *WOX4* is regulated by the repressive mark H3K9me2, while *LEC1* and *BBM1* are epigenetically regulated by H3K27me3. We conclude that epigenetic regulation plays an important role during somatic embryogenic development, and a molecular mechanism for SE is proposed.

## Introduction

All living organisms ensure their species’ survival by reproduction. Both mammals and higher plants use different strategies of reproduction through gamete fusion, through which an embryo is developed. However, plants, unlike mammals, have developed sophisticated strategies of reproduction without egg fertilization to ensure the survival of the offspring [1], such as apomixis or vegetative propagules [2], [3]. For instance, in *Tripsacum*, a weedy relative of maize, the development of an embryo from an egg cell occurs without fertilization [4], and some Agave species can generate small plantlets from inflorescences or rhizomes [5]. Taking advantage of the ability of plants to regenerate a new plant from a cell or group of somatic cells, these organisms have developed a process known as somatic embryogenesis (SE). SE is one of the most intriguing processes in plants [1] and a powerful biotechnological tool to multiply plants that are difficult to propagate by conventional methods or for plants at risk of extinction [6]. There have been several attempts to understand the molecular mechanisms that occur during the transition from somatic cell to embryogenic cell [7], [8], [9].

It has been proposed that genes such as *BABY BOOM1* (*BBM1*) and *LEAFY COTYLEDON1* (*LEC1*) are needed during the beginning of SE [10], [11]. *BBM1* is preferentially expressed in developing embryos and seeds of *Brassica napus*, while *BnBBM1* overexpression promotes cell proliferation and morphogenesis during embryogenesis [10]. In addition, it has been identified that *BBM1* activates genes involved in cell wall modifications associated with dividing and growing cells, suggesting that *BBM1* activates a complex network of developmental pathways associated with cell proliferation [12]. On the other hand, *LEC1* plays a central role in seed maturation in *Arabidopsis,* and it has been proposed as a key regulator for embryogenic identity. It is also thought that its ectopic expression promotes embryo development [11]. Furthermore, *AtLEC1* integrates activities at diverse regulatory levels, such as transcription factors, hormones and light signaling in both somatic and zygotic embryogenesis [13]. All these findings indicate common developmental pathways between somatic and zygotic embryogenesis. Another gene that has been related to SE is the *WUSCHEL-RELATED HOMEOBOX* (*WOX*), which has specialized functions in various developmental processes in plants, such as embryogenic patterning and stem cell maintenance [14]. For instance, in *Arabidopsis* and tomato, *WOX4* plays an essential role promoting and maintaining the vascular procambium [15], [16], while in SE of *Vitis vinifera, WOX4* increases its expression levels when the embryo begins to germinate [17]. Despite such advances in the understanding of the molecular basis of SE, the epigenetic mechanisms, such as DNA methylation and histone modifications, that occur during this important biological process are not well understood [18]–[21].

DNA methylation and histone modifications occur widely during cellular differentiation and development in plants and mammals [18], [22], [23]. DNA methylation is one of the epigenetic regulatory mechanisms most studied in plant development, and the scientific contributions related to its role in blooming, endosperm development, response to stress, genome maintenance, gene silencing, control of transposable elements and genomic imprinting have helped to understand important regulatory processes [24]–[26]. In the case of histone modifications, it is known that they are required in the activation or repression of gene transcription by changing chromatin structure. For instance, di- or trimethylation of histone H3 at lysine 4 and 36 (H3K4me2/me3 and H3K36me2/me3) are related to transcriptional active chromatin [27], [28]; in contrast, H3K9me2 and H3K27me3 are considered to be repressive marks [29]–[31]. Previous reports have shown that embryogenic cell formation increases DNA methylation in *Daucus carota* and *Cucurbita pepo* [32], [33]. However, Charkrabarty et al. [34] reported that in order to obtain an embryogenic calli from *Eleuterococus senticosus,* low levels of DNA methylation are needed. More recently, Viejo et al. [20] showed in *Castanea sativa* that DNA demethylation is required for SE induction and further development of somatic embryos in this species. On the other hand, it was recently reported that both DNA methylation and H3K9me2 modulate *WUSCHEL* expression *in vitro* during shoot regeneration in *Arabidopsis* [35]. Furthermore, the H3K27me3 repressive mark plays an important role in the regulation of genes involved in biosynthesis, transport, perception and signaling of auxins, and zygotic embryogenesis development [36], [37]. Based on this information, the aim of the present study is to further extend our understanding of the epigenetic means by which SE in plants is regulated and propose a regulatory mechanism for *LEC1*, *BBM1* and *WOX4* in somatic embryo development. In this study, we used *Coffea canephora* due to its highly embryogenic response *in vitro* [38] and its economical relevance worldwide. *C. canephora* is one of the two economically important species of coffee and it represents the 25% of coffee in the market. *Coffea* spp is the second most traded commodity in the world after oil. Raw coffee generates between $15 and $20 billion per year for exporting countries [39]. Because of these economic aspects, many research initiatives have been targeted genomic and transcriptomic data of *Coffea* spp., which could contribute to an understanding of the biology of coffee [40], [41]. However, studies in epigenetics are also needed to understand more about the molecular mechanisms of growth and reproduction in this plant. The results of this work reveal specific chromatin modifications during SE in coffee, which could give some answers about the regulatory events that take place during embryo development to improve breeding practices.

## Results

### Induction, Morphology and Histology of Somatic Embryogenesis in *Coffea canephora*

**Figure 1:**
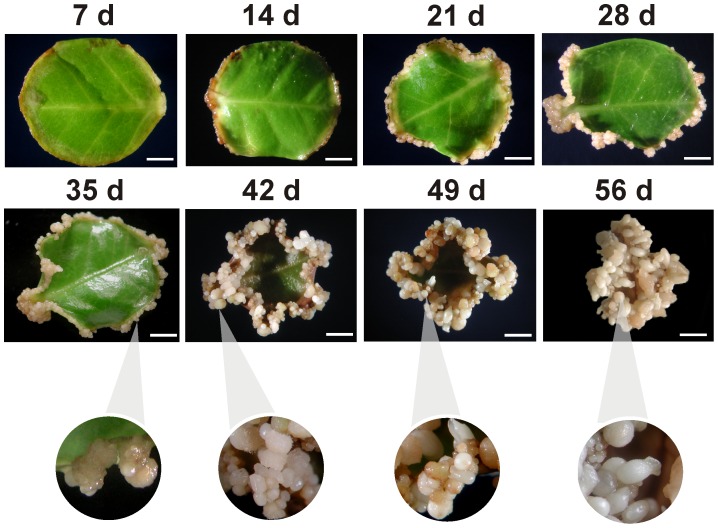
Development of the somatic embryogenesis process in *Coffea canephora*. Leaf explants were cultured in liquid Yasuda medium in the presence of 5 µM of benzyl adenine under dark conditions. The embryogenic process starts with the thickening of leaf edges at 14 days after induction (dai), cellular proliferation and the development of the proembryogenic mass (Pm), between 21 and 28 dai. Differentiation of the first somatic embryogenic structures from Pm starts to appear at 35 dai and the differentiation of the late embryogenic stages begins at 42 dai until 56 dai. A close-up of the embryos is shown in circles. Bars = 4 mm.

**Figure 2:**
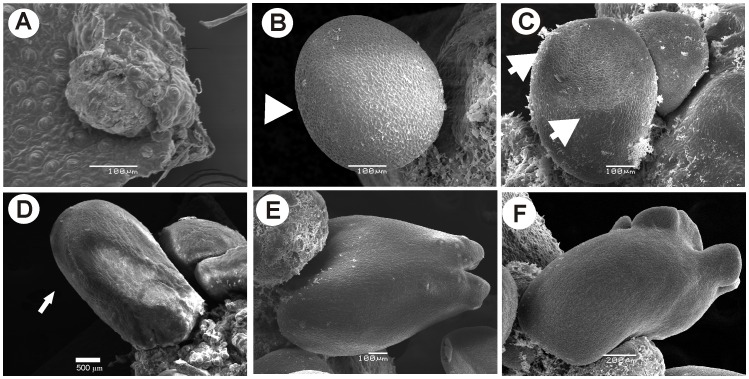
Scanning electron microscopy of the somatic embryo development in *Coffea canephora*. **A)** Proembryogenic mass (Pm) from a leaf explant at 21 days after induction (dai). **B)** Globular stage; the white arrowhead indicates the protoderm establisment. **C)** Heart stage; the white arrows indicate the beginning of the cotyledonary primordia in the embryo. **D)** Torpedo stage; the white arrow indicates the enlargement of the embryo. **E)** Early cotyledonary stage, where the establishment of the future cotyledons can be observed. **F)** Late cotyledonary stage. At this stage the cotyledons are fully developed. Bars = 100 µm.

**Figure 3:**
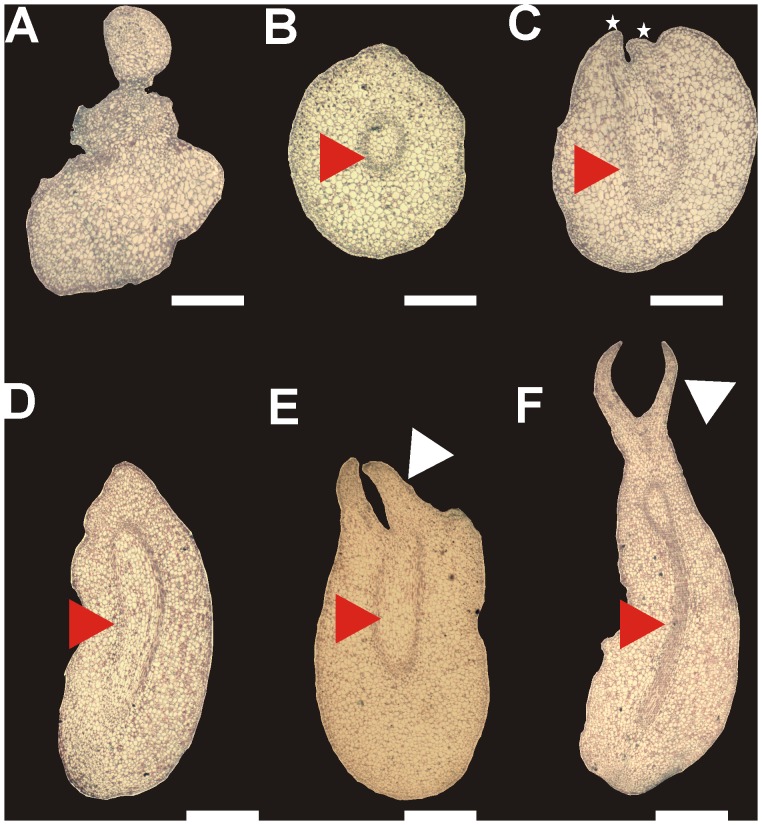
Histological dissection of the somatic embryo at different developmental stages in *Coffea canephora*. **A)** Transverse cut of the early globular stage from the proembryogenic mass at 14 dai. **B)** Transverse cut of the globular stage. The red arrowhead indicates the cellular organization and the presence of a well-defined procambium. **C)** Longitudinal cut of the heart stage. The red arrowhead indicates the initiation of the elongation of the procambium zone and the beginning of the cotyledonary primordium is indicated with white stars. **D)** Longitudinal cut of the torpedo stage. The red arrowhead indicates the elongation of the procambium zone. **E)** Longitudinal cut of the early cotyledonay stage. The red arrowhead indicates the procambium zone, while the white arrowhead indicates the development of the early cotyledonary primordium. **F)** Longitudinal cut of the late cotyledonary stage. The red arrowheads indicate the procambium zone, and the white arrowhead indicates the development of cotyledonary primordium. Bars = 200 µm.

In order to investigate the epigenetic and molecular changes at the different developmental stages of somatic embryo formation, we performed *in vitro* SE induction. *In vitro C. canephora* plantlets were preconditioned with naphthalene acetic acid (NAA) and kinetin for 14 days. Then, young leaves were cut and cultured in liquid medium supplemented with 5 µM benzyl-adenine (BA) for 56 days (see Materials and Methods). Between 21 and 28 days after induction (dai), the dense cellular formation known as proembryogenic mass (Pm) was noticed (Figure 1). The formation of new meristematic centers in Pm allows the emergence of the first embryogenic stage, the globular (G), at day 35 (Figure 1 and Figure 2). After that, the formation of several embryogenic structures such as the G, the heart stage (H), the torpedo stage (T) and the cotyledonary stage (C) were observed at 42 and 49 dai (Figure 1 and Figures 2B–F). Finally, at 56 dai, the explant was surrounded by all embryo stages (Pm, G, H, T and C), and, by scanning electron microscopy, it was observed that Pm emerges from the inner cells in the explant margin (Figure 2). In the case of H, a bifurcation on the top of the structure was observed (Figure 2C). During the transition from H to T, the embryo starts to elongate, and the axis polarity starts to appear; this polarity is crucial for the formation of the apical and radicular meristems (Figure 2D). During the last stage, the C stage, the cotyledons start to expand and separate (Figure 2F). Histological analyses also revealed that new meristematic centers allowing to early G stage, originate from embryogenic cells, which are small with respect to neighboring cells (Figure 3A). In the G stage, the procambium cells, which form the basic structure of the future plant, are well defined (Figure 3B). During the transition from the G to the H stage, the embryogenic axis for elongation and the split of the procambium are defined (Figure 3C). The T stage presents an elongated embryo, and a fully polarized procambium was observed (Figure 3D). Finally, in the C stage, the presence of the cotyledons in the embryo is evident (Figure 3E) and both the shoot and root stem cell pools are well established (Figure 3F).

### Changes in DNA Methylation during Somatic Embryogenesis

**Figure 4:**
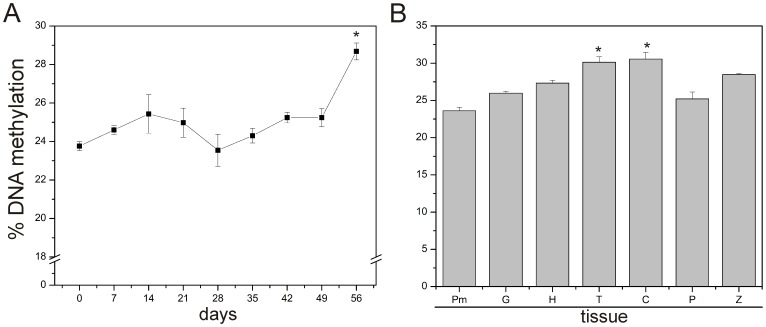
Global DNA methylation levels during somatic embryogenic induction and the separate embryogenic stages and tissues of *Coffea canephora*. **A)** Percentage of global DNA methylation during the development of the somatic embryogenesis process in *Coffea canephora* shown in Figure 1. **B)** DNA methylation levels of different tissues and developmental somatic embryo stages. Pm: Proembryogenic mass, G: Globular stage, H: Heart stage, T: Torpedo stage, C: Cotyledonary stage, P: *C. canephora in vitro* plantlets, Z: *C. canephora* zygotic embryo in cotyledonary stage. Bars represent the mean ± SE (*n* = 3). An asterisk represents the statistical significance of mean differences at a given time by the Tukey test (*P*≤0.05). Each experiment was carried out three times.

In order to know whether DNA methylation plays a role in the SE process, we analyzed global DNA methylation during the SE temporal course of 56 days (Figure 4A) and at different developmental stages of somatic embryos (Figure 4B). It was observed that during the beginning of the 56-day course of the SE process, there is a gradual increase in 5-methyl-2′-deoxycytosine (5 mdC) levels, from 23.8% at the beginning of the induction (0 days) to 29% at 56 days (Figure 4A). However, at days 21 and 28, a decrease in DNA methylation content was observed, 24.8% and 23.5%, respectively (Figure 4A). This might be related to a rapid cell proliferation of the dedifferentiated tissue (Figure 1; 28 days). However, by day 35, when the first embryo stages begin to appear, a gradual increase in the level of DNA methylation was observed until day 56, when a significant increase (indicated with an asterisk) in DNA methylation was observed (Figure 4A). These results indicate that the differentiation of the embryogenic structures is accompanied by an increase in DNA methylation (Figures 1 to 4).

Since we observed important changes in the content of DNA methylation during the course of SE development (Figure 4A), we analyzed the content of DNA methylation in each embryo developmental stage (Figure 4B). Somatic embryos were separated by stages (Pm, G, H, T and C) and their DNA methylation content was compared with that of the *in vitro* plantlet (P) and the zygotic embryo, in its cotyledonary stage, isolated from coffee seeds (Z) (Figure 4B). The lowest DNA methylation percentage (23.7%) was observed in Pm, which was separated from the explant and isolated at 28 dai. It was also observed that DNA methylation increases as the embryo develops, and the highest content of DNA methylation was found in the T and C stage. On the other hand, the plantlets presented a difference of 5% in DNA methylation content in comparison with the somatic cotyledonary stage (Figure 4B), whereas the zygotic embryo had 2% less DNA methylation than the somatic embryo at the same developmental stage (Figure 4B).

### Effect of 5-Azacytidine during the Somatic Embryogenic Process

**Figure 5:**
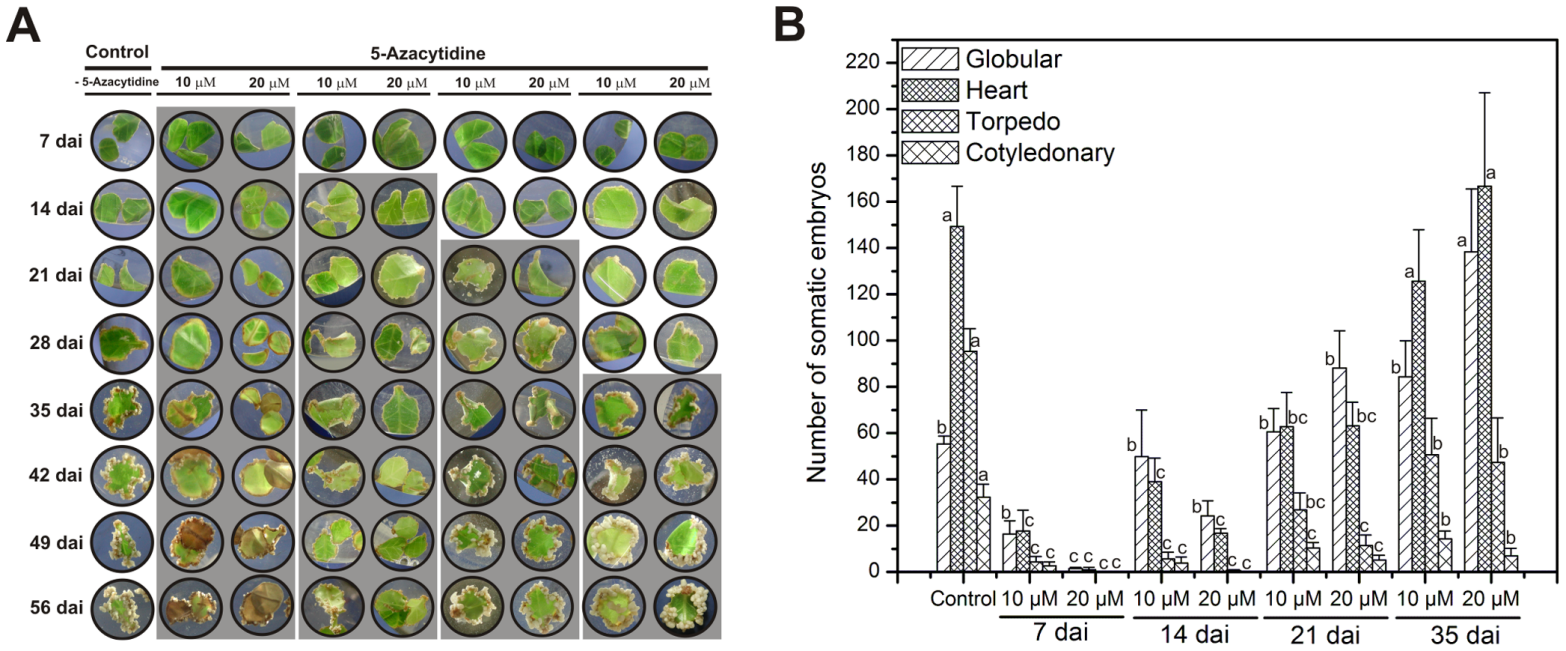
Effects of 5-Azacytidine (5-AzaC) in the somatic embryogenesis of *Coffea canephora*. **A)** Temporal course of explants from *C. canephora* leaves under embryogenic induction with and without (control) the presence of 5-AzaC at 10 µM and 20 µM. The 5-AzaC was added into the medium every seven days starting at different independent time points (day 7, 14, 21 and 35) of the SE culture. The shaded rectangles mean the presence of 5-AzaC in the medium, as indicated in Materials and Methods. **B)** The number of every somatic embryo stage was counted after 56 days after induction (dai), with and without (control) the treatments with 5-AzaC added at different time points after the induction (day 7, 14, 21 and 35). The bars represent the mean ± SE (*n* = 3). Different letters in columns represent the statistical significance of mean differences between each embryogenic stage at a given time by Tukey test (*P*≤0.05). The experiments were performed three independent times.

In order to know whether the increase in DNA methylation is related to the onset and differentiation of somatic embryos, a pharmacological assay was performed with two different concentrations (10 µM and 20 µM) of 5-azacytidine (5-AzaC, a DNA methylation inhibitor) and without 5-AzaC (control) (Figure 5). The 5-AzaC was added every 7 days (until day 56) starting at independent time points (7, 14, 21 and 35) to see the effect of this compound when it is added at the beginning (day 7 or 14) or at the end of the process (day 21 or 35). The number of somatic embryos from each developmental stage in each of the four time experiments with 5-AzaC was counted at 56 dai (Figure 5B; see Materials and Methods). It was observed that 5-AzaC had a dramatic negative effect on the embryogenic response when it was added from day 7 after induction at both concentrations. However, this negative effect was not observed when this inhibitor was added at day 35 after induction (Figure 5A). Furthermore, it was observed that 5-AzaC at 10 µM, added at day 7, provoked a reduction of 86% in the total number of somatic embryos and at 20 µM a reduction of up to 98%, in comparison with the control without 5-AzaC (Figure 5B). Interestingly, the effects of 5-AzaC when it was added at day 14 were less dramatic in comparison with its addition at day 7 (Figure 5). On the other hand, we did not observe visible impairing effects due to 5-AzaC in the formation of the somatic embryo in both concentrations added at day 21; in contrast, it was found that its presence increases the proliferation of Pm, delaying the formation of embryogenic structures (Figure 5A). However, the presence of 20 µM of 5-AzaC increases the number of G embryos by 1.6 fold in comparison with the control (Figure 5B). This result suggests that the effect of 5-AzaC (mainly at 20 µM) added at day 21 after induction, not only seems to synchronize the embryogenic process, but also reduces the embryo maturation (Figure 5). Furthermore, this result was also observed in the treatment with 5-AzaC at day 35, when we observed higher somatic embryos at early stages of development, mainly G and H, in comparison with the control. These findings suggest that 5-AzaC can disrupt the normal development of somatic embryos at early stages of the process, probably by affecting threshold levels of DNA methylation. Therefore, to test this hypothesis, we assessed whether impaired-embryo formation due to 5-AzaC was indeed due to a loss in DNA methylation levels. The explants from day 7 were treated with 10 µM of 5-AzaC, every seven days from day 7 until day 56, and the content of 5 mdC was evaluated (Figure S1). The results show that the addition of 5-AzaC drastically reduces the content of DNA methylation from 23.5% at 7 dai to 14% at 56 dai (Figure S1). These results indicate that gradual DNA demethylation due to the addition of 5-AzaC (10 µM) from day 7 after induction is directly correlated with impaired embryogenic induction (Figure 5).

### Histone Methylation Patterns during SE

**Figure 6:**
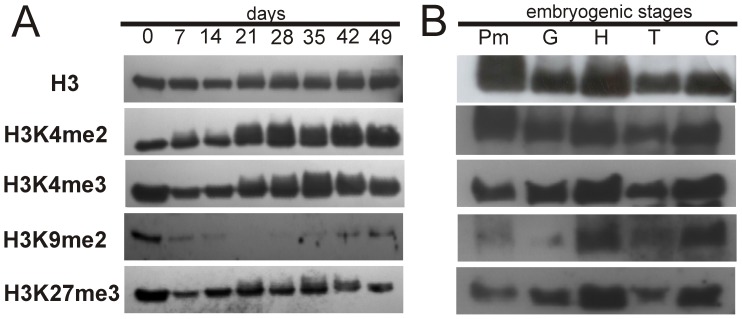
Histone H3 methylation patterns during somatic embryogenesis induction and in different embryogenic stages of *Coffea canephora*. **A)** Immunoblot analyses during the development of the somatic embryogenesis process in *Coffea canephora* shown in Figure 1. **B)** Immunoblot analyses of different tissues and developmental somatic embryo stages. Total histone extracted from leaf explants, as shown in Figure 1, from 0 until 49 days after induction and from different somatic embryo developmental stages were probed with specific antibodies in Western blots. Subsequent to the hybridization, membranes were stripped off and re-probed with antibodies specific to non-modified histone H3. Pm: proembryogenic mass, G: globular stage, H: heart stage, T: torpedo stage, C: cotyledonary stage.

It is known that histone posttranslational modification is a fundamental key for chromatin conformation and regulation of transcriptional activity of genes related to development [42], [43]. Therefore, we investigated whether, besides DNA methylation, global histone H3 methylation is related to the embryogenic response and establishment of the somatic embryo in *C. canephora* (Figure 6). Nuclear proteins were isolated from explants under embryogenic induction in a temporal course of 49 days (Figure 6A; see Materials and Methods) and from somatic embryos from each developmental stage (Figure 6B). Changes in H3 methylation were detected by Western blot using antibodies against di- and trimethylation of H3K4, H3K9me2 and H3K27me3. We observed interesting changes in global histone methylation patterns in the explants under embryogenic conditions. For instance, after embryogenic induction (day 7), a decrease in the global H3K4me3, H3K9me2 and H3K27me3 marks compared with day 0 was observed (Figure 6A). Interestingly, the presence of the repressive mark H3K9me2 was undetectable at 21 and 28 dai, whereas a decrease in DNA methylation was also observed on these days during the SE process (Figure 4A). On the other hand, the H3K27me3, another repressive mark, was maintained unchanged to the end of the process, an exception for day 7 (Figure 6A).

In order to know whether these epigenetic marks in the H3 histone were changed in each developmental stage of the embryo, the global histone H3 methylation patterns of H3K4me2, H3K4me3, H3K9me2 and H3K27me3 from Pm to C stages were evaluated (Figure 6B). We detected dynamic changes in the global H3K9me2 and H3K27me3 marks and particularly in the Pm, G and T stages were low. Interestingly, these repressive marks are increased in the H and C stage. On the other hand, the H3K4me2 and H3K4me3 marks were abundant throughout all somatic embryo stages. All together, the results indicate that global histone methylation changes (especially both repressive marks H3K9me2 and H3K27me3) together with DNA methylation (Figure 4) could contribute to the transition from somatic cells into somatic embryos.

### Gene Expression Patterns during the SE

Previous reports have shown that *LEC1* and *BBM1* play a crucial role during the SE process [7], [10], [11]. Therefore, we searched these genes in *C. canephora* in the GenBank (http://www.ncbi.nlm.nih.gov/UniGene/library.cgi?ORG=Cca&LID=25442) and in the Sol Genomics Network (http://solgenomics.net/content/coffee.pl). We found that the ORF of the sequence GT656663.1 of *C. canephora* encodes for the central B domain of the HAP3 subunit of the transcription factor LEC1, required for DNA binding [11], which has a high degree of similarity to the B domains of the other LEC1 orthologs (Figure S2). For instance, this sequence showed 82% similarity to both *Arabidopsis thaliana* (*At*LEC1) and *Medicago truncatula* (*Mt*LEC1), 83% to *Daucus carota* (*Dc*LEC1), 84% to *Isoetes sinensis (Is*CAAAT-box), 86% to both *Brassica napus* (*Bn*LEC1) and *Pistacia chinensis (Pc*LEC1), 87% to both *Zea mays* (*Zm*LEC1) and *Oryza sativa (Os*LEC1) and 95% to the sequence of *Theobroma cacao* (*Tc*LEC1). On the other hand, we found that translation products of the sequences GT656313.1 and GT656297.1 of *C. canephora* show a high degree of similarity to the double APETALA2/ETHYLENE RESPONSE FACTOR (AP2/ERF) DNA-binding domains of the transcription factor BBM1 (Figure S3) according to Boutilier et al. [10]. For instance, the alignment of these sequences with other orthologs of BBM1 has 95% similarity to *Vitis vinifera* (*Vv*BBM1), 92% to *Tc*BBM1, 91% to *Os*BBM1, 86% to *Bn*BBM1, 85% to both *Glycine max* (*Gm*BBM1) and *At*BBM1, 82% to *Mt*BBM1 and 81% to *Zm*BBM1.

On the other hand, it has been shown that WOX4 functions to promote differentiation of the vascular procambium [16], but its expression has been observed principally during postembryogenic development or during germination [17]. However, its role during SE and its regulation is unclear. Therefore, we used the SGN-U627534 sequence of *C. canephora,* which contains the homeodomain of WOX4 (Figure S4), which at the amino acid level has a high degree of similarity with respect to other orthologs: 100% similarity to *Solanum lycopersicum* (*Sl*WOX4), 99% to *Gm*WOX4, 97% to *Vv*WOX4, 96% to *At*WOX4, 94% to *Populus trichocarpa* (*Pt*WOX4), 83% to *Os*WOX4, 82% to *Zm*WOX4 and 80% to *Brachypodium distachyon* (*Bd*WOX4). The WOX homeodomain has been found to bind to DNA through a helix turn helix structure [14]. Therefore, we used the *C. canephora*-conserved sequences to perform the expression analysis of *LEC1*, *BBM1* and *WOX4* in different embryogenic developmental stages.

**Figure 7:**
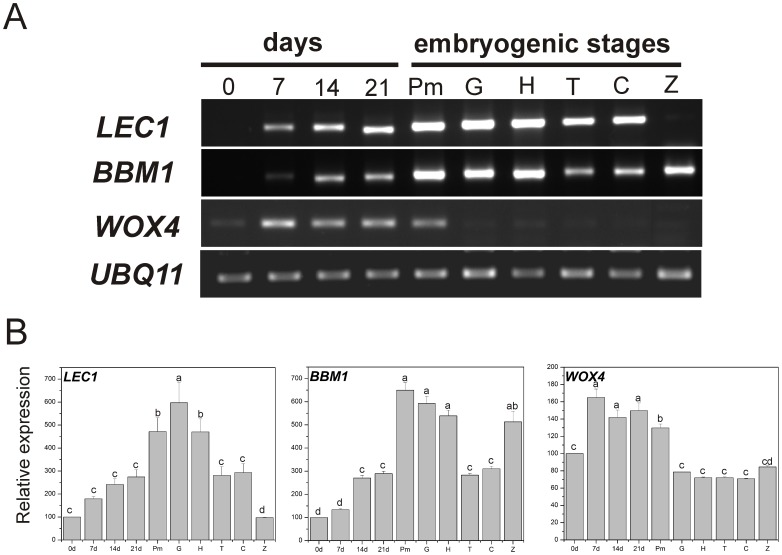
Expression profiles of genes during embryogenic induction and development of the somatic embryos in *Coffea canephora*. **A)** Expression of *LEC1, BBM1* and *WOX4* was performed from total RNA samples for RT-PCR analysis that were isolated from leaf explants under embryogenic induction at days 0, 7, 14 and 21, and the different embryogenic stages were isolated and comparatively classified in proembryogenic mass (Pm), globular (G), heart (H), torpedo (T), cotyledonary (C) and zygotic embryo (Z) in cotyledonary stage. *UBQ11* was used as a reference gene. **B)** Densitometric analysis of the gene expression showed in **A**. Relative expression of *LEC1, BBM1* and *WOX4* were normalized to the constitutive gene *UBQ11*. Different letters in columns represent the statistical significance of mean differences at a given time according to the Tukey test (*P*≤0.05). Each RT-PCR was conducted twice with three independent biological replicates.

The relative expressions of *LEC1*, *BBM1* and *WOX4* genes were evaluated by RT-PCR assays during embryogenic induction (0, 7, 14, and 21 dai), as well as at the different embryogenic stages (Pm, G, H, T, C and Z) (Figure 7). We found that all genes were absent or expressed at low levels on day 0, and the only gene that was highly expressed in the zygotic embryo (Z) was *BBM1*. In the case of *BBM1*, the highest expression was found in the embryogenic stages of Pm, G and H, while in the more developed stages, such as T and C, the expression of these genes was reduced. During the 21 days of the SE induction process, *BBM1* expression was low with respect to that found during the somatic embryo development (Figure 7B). These results suggest that the expression of *LEC1* and *BBM1* genes are important for differentiation and maturation of the embryos. *BBM1* activates pathways related to cell proliferation and growth, while *LEC1* is required to induce the embryogenic program and embryo maturation [10], [11]. On the other hand, the expression of *WOX4* was almost undetectable in the different embryogenic stages, and its expression was only found at days 0, 7, 14, 21 and in Pm (Figure 7). *WOX4* participation during SE has not been studied in detail in other species, but during the germination of somatic embryos of *V. vinifera,* the *WOX4* transcripts are higher [17]. In addition, our result regarding the lack of expression of *LEC1* in Z are consistent with other studies in *Arabidopsis* and *B. napus* [11], [44], suggesting that LEC1 cannot be involved in the postembryogenic regulation due to the fact that LEC1 plays a more central role in embryo development [11], [45].

### Epigenetic Regulation of *LEC1*, *BBM1* and *WOX4* during SE

**Figure 8:**
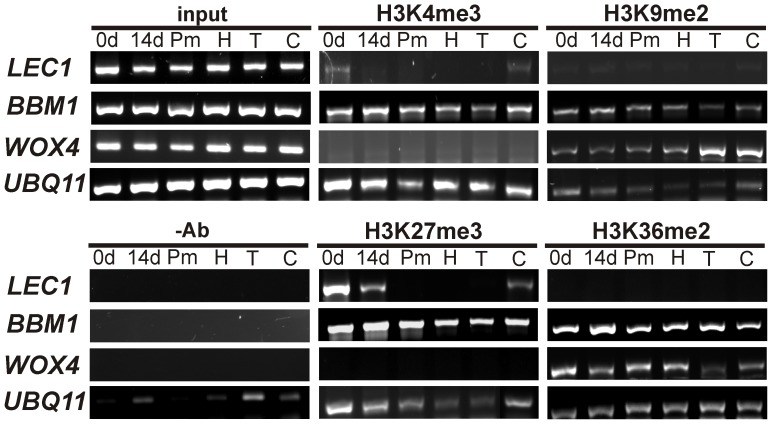
Histone H3-methylation patterns during somatic embryogenesis development in *Coffea canephora* using Chromatin Immunoprecipitation (ChIP). Samples were collected at the beginning of embryogenic induction (0d) and 14 days after embryogenic induction (14d) and during the proembryogenic mass (Pm), torpedo stage (T) and cotyledonary stage (C). The samples were examined for the Histone H3-tail methylation patterns, and the *LEC1*, *BBM1* and *WOX4* genes. Input (Input DNA): 10-fold diluted samples were used as templates for the input lanes. As negative control (-Ab), no antibody samples were treated in the same way as immunoprecipitated chromatin with H3K4me3, H3K9me2, H3K27me3 and H3K36me2. Amplified *UBQ11* with specific primers was used as the control for the quality of samples and the same amounts was used to amplify *LEC1, BBM1* and *WOX4*.

Because *LEC1*, *BBM1* and *WOX4* showed differential expression at the beginning of the SE process and at different embryogenic stages (Figure 7), we examined the epigenetic marks of histone H3-methylation (H3K4me3, H3K9me2, H3K27me3 and H3K36me2) in these genes by Chromatin Immunoprecipitation (ChIP) at days 0 and 14 and in the embryo developmental stages Pm, H, T and C (Figure 8). We did not observe an enrichment of the marks related to gene expression of H3K4me3 and H3K36me2 in any of the genes evaluated. However, we found that the H3K9me2 mark was accumulated from the H to the C stages in the sequence that encodes for the homeodomain of the *WOX4* gene (Figure S4), which is important for DNA binding. This mark accumulation seems to be related to the lack of *WOX4* gene expression found in Figure 7, while the presence of H3K36me2 from 0 to Pm suggests its participation during the expression of this gene.

In the case of the H3K27me3 mark, it was observed that *LEC1* and *BBM1* genes were enriched with this repressive mark at different embryo stages. For instance, in *LEC1* this mark was highly enriched at 0 days and present only slightly at both 14 days and the C stage (Figure 8); this result is related with the repression found of this gene in those days (Figure 7). Similar results were found for *BBM1*, in which we found that the genomic region that codified for the repeat 2 AP2/ERF domain (Figure S3), which is a fragment needed for DNA binding [10], is highly marked with H3K27me3, especially at the beginning of induction (0 to 14 days). Furthermore, we observed that this region carries a moderate level of H3K4me3 and H3K36me2 marks in all tissue, but low levels of H3K9me2. This result suggests that the decrease of H3K27me3, and the presence of H3K4me3 and H3K36me2, might favor the transcription of *BBM1* from Pm to C stage (Figure 7). Overall, *WOX4*, *LEC1* and *BBM* expression are regulated by histone modifications.

### 5-Azacytidine Affects the Expression of *LEC1* and *BBM1*

**Figure 9:**
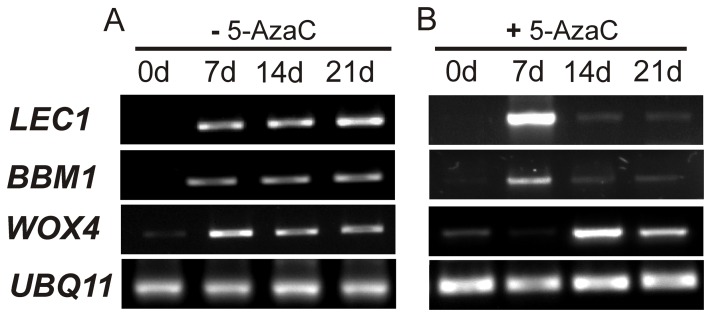
Effects of 5-Azacytidine on the *LEC1, BBM1* and *WOX4* expression during the beginning of somatic embryogenesis in *Coffea canephora*. **A)** Gene expression under embryogenic conditions in the absence of 5-Azacytidine. **B)** Gene expression under embryogenic conditions with 10 µM 5-Azacytidine added every seven days from 0 to 21 dai. Total RNA was used for RT-PCR analysis. *UBQ11* was used as a reference gene.

Because we found that DNA demethylation generated by 5-AzaC arrests the somatic embryogenic process (Figure 5), we assessed whether this demethylating agent also affects the transcription of *LEC1* and*BBM1*, which are important to induce the embryogenic program and the morphogenesis from somatic cells [10], [11], as well as *WOX4* during the initiation of SE. For this purpose, the explants of coffee were incubated in the presence of 10 µM of 5-AzaC, added every seven days from day 0 until 21 dai (Figure 9). It was found that all three genes, *LEC1*, *BBM1* and *WOX4*, under normal embryogenic conditions (Figure 9A, without 5-AzaC), are expressed almost at the same level from day 7 until 21 dai. However, in the presence of 5-AzaC, *LEC1* is highly expressed at day 7 and is low or almost undetectable at days 14 and 21 (Figure 9B). In the case of *BBM1,* this gene was expressed at the same level as without 5-AzaC at day 7 but its expression was almost undetectable at days 14 and 21. On the other hand, *WOX4* expression was increased with 5-AzaC at days 14 and 21 but was undetectable at day 7. Although all together these results show differential regulation in *LEC1*, *BBM1* and *WOX4* in the presence of 5-AzaC, we cannot rule out the possibility of second effect of cell death observed in explants treated with this demethylating agent (Figure 5A). Further studies need to be done in order to know whether 5-AzaC is activating the expression of *LEC1* (at day 7) and *WOX4* (at days 14 and 21) by indirect epigenetic mechanisms due to the activation of histone methyltransferases.

## Discussion

The capacity of somatic cells to form somatic embryos and regenerate a new plant is known as somatic embryogenesis (SE) [46]. Although SE has been studied for a long time, the process is not fully understood, and the importance of epigenetic mechanisms during SE and in different embryogenic stages has not been addressed. The results presented here provide new insight into the epigenetic regulation needed during the SE of one of the most economically important species of coffee, *Coffea canephora*.

In this study, we showed that during the beginning of SE in *C. canephora* (Figure 1), the cellular differentiation process and embryo development are modulated by epigenetic mechanisms such as DNA methylation (Figure 4) and histone methylation (Figure 6). For instance, we observed that the explants of *C. canephora* at day 0 have 23.8% global DNA methylation, while at the beginning of the differentiation process it increased to 25.4% (day 14, Figure 4A). This strongly suggests a rapid cellular response to *in vitro* conditions [47], [48] accompanied by a drastic chromatin remodeling [49], [50]. Previous reports have shown that embryogenic cell formation is related to the increase of DNA methylation [20], [32], [33]. However, an increase in DNA methylation is not always the condition that has been reported to be important for SE; a decrease in DNA methylation levels seems to be fundamental for embryo development in some species. For instance, during the phase of dedifferentiation or embryogenic calli generation in *E. senticosus* and *Rosa hybrida,* DNA demethylation events are frequent [34], [51]. Recent studies in *C. sativa* and *Acca sellowiana* have also showed the importance of demethylation events during SE induction or prior to the start of embryo differentiation [20], [52]. We observed two increases in DNA methylation during the SE of *C. canephora* (Figure 4A). The first was observed from day 7 until day 21, when we observed the stronger effect of 5-AzaC on SE induction (Figure 5), and the second increase was observed at the end of the SE process, from day 35 until day 56 (Figure 4A). This suggests that there is a DNA methylation dynamic throughout the whole process of embryo formation, confirming the important role of DNA methylation patterns during the development of plants [53]–[55]. Besides the changes in DNA methylation observed during SE development (Figure 4A), we found interesting changes in different embryo developmental stages (Figure 4B). For instance, we found that somatic embryos in the cotyledonary stage contain high levels of DNA methylation, while the zygotic embryo, at its cotyledonary stage, presented lower levels of DNA methylation (Figure 4B), which can be related to the arrest of development during the dormant period that the zygotic embryo suffers inside the seed [56]. In contrast, the embryo in its C stage continues with the development.

Previous reports have shown that DNA methylation patterns are involved in the control of diverse phases of development in both plants and animals [23], [54]. To assess that DNA methylation is important during the SE of *C. canephora,* we performed pharmacological studies to evaluate the effect of 5-AzaC during the SE process (Figure 5). We found that 5-AzaC added since 7 dai drastically reduced the SE process, and this compound induces DNA hypomethylation (Figure S1). Fraga et al. [52] showed that during SE induction in *A. sellowiana,* the levels of DNA methylation increase even in the presence of 5-AzaC. However, the presence of this compound resulted in a lower conversion of embryos into plantlets. On the other hand, in *Medicago truncatula*, it was observed that the use of 5-AzaC causes the loss of SE through DNA demethylation [57], a result which agrees with those found in our studies. Therefore, it seems that DNA methylation plays an important role in embryo formation in model and non-model plants. On the other hand, we detected that the effects of 5-AzaC were reduced depending on the day that this compound was added (Figure 5B). For instance, we observed that when 5-AzaC was added at 7 or 14 dai, the SE was drastically affected. However, it is worth noting that 5-AzaC seems to synchronize the earliest stages of embryo development and to reduce embryo maturation (Figure 5). Similar results have been observed in *D. carota*, where 5-AzaC arrests the development of the H stage, inducing secondary embryogenesis [58]. On the other hand, Yamamoto et al. [32] reported that the effects of this demethylating agent depend on the embryogenic stage at which it is applied. The SE in *D. carota* is arrested whether it is applied at 3 or 7 dai, but there is no difference in the embryogenic response if it is applied from 7 to14 dai. It has been also found that the presence of 5-AzaC or the use of the *METHYLTRANSFERASE1* (*met1*) mutant increases the formation of meristematic centers improving the shoot formation from calli of *Arabidopsis* [35], [59], indicating that DNA methylation plays a role mediating the development rate. However, although many reports have pointed out that DNA methylation is involved in embryo and plant development, the mechanism through which it happens is still unknown. Most likely, hypomethylation in the whole genome due to the effect of 5-AzaC is turning on genes that need to be repressed in a specific developmental time sequence in order to induce embryo maturation. The specific inhibitory methylation effect of 5-AzaC needs to be studied in order to test this hypothesis.

Besides the changes in DNA methylation observed during the SE process (Figure 4), we found an interesting histone methylation pattern (Figure 6) that seems to be related to the reduction in DNA methylation levels at 21 and 28 dai (Figure 4A). We observed an absence of the repressive mark H3K9me2 and an increase of the transcription-related marks H3K4me2 and H3K4me3 (Figure 6A); on the same days, a reduction on DNA methylation was observed. Similar epigenetic events have previously been shown in mammalian development during early embryo onset in the zygote, where a significant loss of DNA methylation and H3K9me2 occurs [22], [60]. Interestingly, we also found a reduced level of H3K9me2 and H3K27me3 marks after embryogenic induction at day 7 (Figure 6A). It has been reported that a decrease in DNA methylation and low levels of H3K9me2 and H3K27me3 allow the expression of genes related to the beginning of cell dedifferentiation [18], [36]. The H3K9me2 mark has been shown to be involved in heterochromatin formation; it is dependent on DNA methylation in *Arabidopsis* and rice [29], [61]. In addition, H3K9me2 contributes actively to the setting up of dedifferentiated states or reentry to the cell cycle [18]. Unlike H3K9me2, a recent report indicates that H3K27me3 controls the expression of ∼9,006 genes in *Arabidopsis* [37], some of which are related to cell differentiation and stem cell regulation. On the other hand, it was found that during the transition from the globular stage to the heart stage there was an increase observed in the repressive marks H3K9me2 and H3K27me3 (Figure 6B), a finding that also seems to be related to an increase in DNA methylation found in the same embryogenic stages (Figure 4B). It has been documented that an increase in DNA methylation is necessary to change the transcription patterns of genes, since DNA methylation represses the transcription directly by interfering with the accessibility of transcription factors [25], [42].

In this study, we have found that two transcription factor genes, *LEC1* and *BBM,* are expressed in different embryogenic stages (Figure 7) possibly by H3K27me3 (Figure 8). This epigenetic mark directly represses only specific transcription factor families, such as HAP3-like and AP2-like transcription factors [37], that also correspond to the genes investigated in this study, *LEC1* and *BBM1,* respectively. *LEC1* is a regulator master of embryogenesis and its expression is needed to induce SE [11], while *BBM1* is essential for cell proliferation and morphogenesis during embryogenesis [10]. We found that analyzed regions of *LEC1* and *BBM* chromatin in coffee are enriched by H3K27me3, which is an epigenetic repressive mark, localized principally to euchromatin regions in plants [62], [63]. We localized the target regions of *C. canephora* in the same genes from epigenomes available from *Arabidopsis* and rice (http://epigara.biologie.ens.fr/index.html and http://www.ricemap.org/gmap.jsp, respectively) (Figures S5 and S6). It was found that, in the case of *AtLEC1*, an important enrichment of H3K27me3 exists in the targeted zone (Figure S5B), which is consistent with our findings (Figure 8). Our results show that the decrease of H3K27me3 in the sequence that encodes for the B-domain of *LEC1,* could be important for its transcription, because it has been observed that the removal of H3K27me3 is important in the temporal control of gene activation [64]. Lee et al. [45] previously showed that the B-domain of *LEC1* in *Arabidopsis* is required for embryogenesis development. These same authors found that the substitution of asparagine 55 by lysine in the B-domain severely decreases the recovery of viable seedlings, suggesting that this amino acid residue is critically required for LEC1 function. Interestingly, the absence of H3K27me3 in *LEC1*, especially during the transition from the H stage to the T stage (Figure 8), means that *LEC1* could be involved in hypocotyl elongation during embryo growth [13].

In the case of *BBM1*, our results show that the second AP2/ERF domain carries high levels of H3K27me3, moderate levels of H3K4me3 and H3K36me2 and low levels of H3K9me2 in all tissues (Figure 8). The comparative epigenetic analysis in *Arabidopsis* (Figure S5) as well as rice (Figure S6) revealed that the targeted region in *BBM1* contains moderated levels of H3K27me3 and low levels of H3K4me3 in both plants. Recently, it has been found in plants that a small group of genes, particularly transcription factors, are marked by both H3K4me3 and H3K27me3 [36]. The same authors suggest that the presence of both antagonistic marks could maintain the repressed transcription status in the genes, but under the differentiation process the balance of these marks could allow rapid transcriptional reactivation. Analysis of *BBM1* overexpression in *Arabidopsis* and tobacco showed that the *BBM1* gene promotes SE even in absence of growth regulators, and induces shoot organogenesis, respectively, suggesting that *BBM1* has the capacity to induce shoot meristem activity as well as embryogenesis depending on the genetic and cellular environment in the cells [10], [65].

On the other hand, it is known that some of the *WOX* gene family members are involved in the regulation of embryogenic cells and maintain meristematic cells, but also they are involved in the regulation of embryo polarity [14], [66]. *WUSCHEL,* a member of *WOX* gene family, which organizes the stem cells in the shoot meristem [67], is modulated by DNA methylation and H3K9me2 in *Arabidopsis* [35]. Here we show that the transcriptional activity of *WOX4* is epigenetically modulated by H3K9me2 (Figure 8), suggesting that another *WUS* homeobox is also controlled through epigenetic mechanisms. It is worth noting that the deposition of H3K9me2 in the *WOX4* gene occurred mainly during embryo elongation, from the H to the C stages (Figure 8). Furthermore, we detected that from the H to the C stage, DNA methylation (Figure 4B) as well as H3K9me2 levels increased (Figure 6B). We also found that during the transition from the H to the C stage, a split of the vascular procambium occurs (Figure 3). Vascular procambium is a group of meristematic cells located at the periphery of stems and roots that are related to the secondary growth [68]. Therefore, taken together, these results indicate that the *WOX4* repression found in these stages (C and H), probably by H3K9me2, is a key step in allowing embryo axis elongation. In *Arabidopsis* and tomato, it has been found that *WOX4* expression is required to promote procambium differentiation in order to regulate lateral plant growth [15], [16].

**Figure 10:**
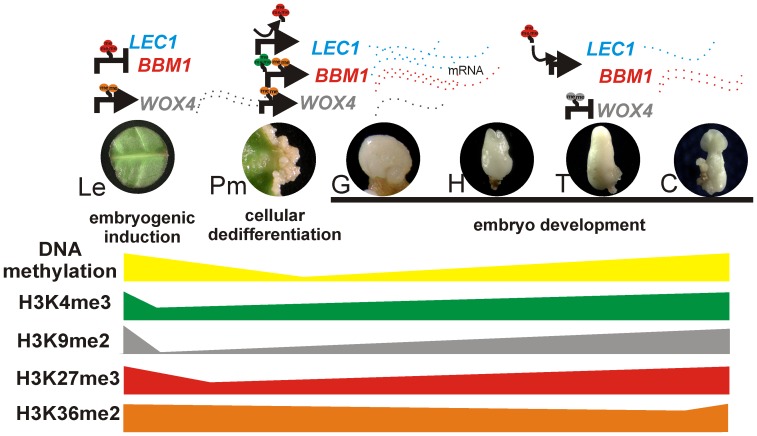
Proposed epigenetic regulatory mechanism during the somatic embryogenesis of *Coffea canephora*. Differentiated somatic cells from leaf explants are treated with plant growth regulators to induce somatic embryogenesis (SE). The embryogenic response proceeds through dynamic changes in DNA methylation and histone modification, each in turn contributing to epigenetic regulation of *LEC*, *BBM1* and *WOX4* genes. Under ideal somatic embryogenic induction, differentiated somatic cells from leaf explants (Le) initiate the first molecular and epigenetic changes. These changes start with the repression of *LEC1* and *BBM1* genes during the induction process, mainly by the accumulation of H3K27me3. *WOX4* is highly expressed in this embryogenic stage, probably by the accumulation of H3K36me2 and the absence of H3K9me2. Furthermore, high levels of DNA methylation are observed. During the Pm development, the H3K27me3 mark on *LEC1* loci is removed and the gene is expressed, while the expression of *BBM1* is mainly accompanied by the accumulation of H3K4me3 and H3K36me2. At this stage, DNA methylation levels start to rapidly decrease. Finally, during the late developmental embryo stage, T, an increase of H3K9me2 promotes the transcriptional repression of *WOX4* and H3K27me3 again starts accumulating on *LEC1* and the expression of *BBM1* decreases. At this embryogenic stage, high levels of DNA methylation are established. These findings suggest that dynamic changes in chromatin could be a crucial step for switching genes on or off during the dedifferentiation and differentiation events to develop a somatic embryo. Le: leaf explant; mRNA: messenger RNA; SE: somatic embryogenesis; Pm: proembryogenic mass; G: globular stage; H: heart stage; T: torpedo stage; C: cotyledonary stage. The arrows mean gene expression while the truncate lines mean gene repression. The abundance of the dashed lines means the abundance of the transcripts.

In summary, we showed that under embryogenic conditions, the somatic cells can be reprogrammed epigenetically through dynamic changes in DNA methylation and histone modifications to promote the embryogenic pathway and development of somatic embryos in *C. canephora* (Figure 10). Our results strongly suggest that a decrease in DNA methylation and reductions of repressive marks H3K9me2 and H3K27me3 could be key steps in triggering the cellular dedifferentiation to acquire cell totipotency, whereas the resetting of these marks seems to be a regulatory mechanism for proper embryo development. The regulation of *LEC1* and *BBM1* expression by the H3K27me3 mark, together with the repression of *WOX4* by H3K9me2, supports the idea that epigenetic mechanisms contribute to the control of the onset and embryo development during SE of *C. canephora.*

## Materials and Methods

### Plantlets, Embryogenic Induction and Growth Conditions

*Coffea canephora* plants were cultivated in Murashige & Skoog [69] medium supplemented with 29.6 µM thiamine-HCl, 550 µM myo-inositol, 0.15 µM cysteine, 16.24 µM nicotinic acid, 87.64 mM sucrose and 0.25% (w/v) gelrite, pH 5.8 and cultured at 25±2°C under a standard photoperiod of 16/8h (150 µmol m^−2^ s^−1^). For the embryogenic induction, the plantlets were transferred to the same medium supplemented with 0.54 µM naphthalene acetic acid and 2.32 µM kinetin for 14 days under the same conditions. Plantlet leaves were cut and five explants of 0.25 cm^2^ were placed on liquid medium (modified Yasuda) as previously described [38] in the presence of 5 µM 6-benzyl-adenine and cultured at 25±2°C under dark conditions at 55 rpm. The plantlets were obtained from three-month-old cotyledonary somatic embryos.

### Electron Microscopy

Somatic embryos at different embryogenic stages (proembryogenic mass, globular, heart, torpedo and cotyledonary) were fixed in phosphate buffer at pH 7.3 (2 mM sodium phosphate monobasic, 2 mM sodium phosphate dibasic heptahydrate and 2.5% glutaraldehyde). Vacuum was applied for 10 min and the culture was maintained at room temperature for 24 h and then kept at 4°C, washing twice with the same buffer without glutaraldehyde. The fixed tissues were dehydrated in a graded series of 10, 30, 50, 70, 85, 96 and 100% ethanol, vacuum was applied at each step for 10 min and the whole maintained for 1 h at 4°C (twice). Then the samples were gradually dried to the critical point with CO_2_ using the dryer Samdri-PVT, and later were mounted on a metallic grill (Polaron SEM coating system E S100) and plated with gold using 30 mA for 60 seconds at 120 mTorr, until a layer of 150 Å was reached. The samples were observed using a scanning electronic microscope (GEOL JSM 6360 LV). Images were obtained by projecting the images at angles of +8° and +8° from the optical axis.

### Histology

Somatic embryos at different embryogenic stages were isolated and fixed in FAA solution [10% formaldehyde, 5% acetic acid 50% ethanol (v/v)] for 48 h and washed five times with phosphate buffer at pH 7.3 (2 mM sodium phosphate monobasic, 2 mM sodium phosphate dibasic heptahydrate). The samples were dehydrated in a graded series of 10, 30, 50, 70, 85, 96 and 100% ethanol and vacuum was applied at each step for 10 minutes and the whole maintained for 1 h at 4°C (twice). Then the samples were embedded in JB-4 resin (JB-4Embedding kit, Polysciences). The blocks were sectioned into 5-µm slices using a MICROM® HM 325 and were double stained with a solution of periodic acid and Schiff’s reactive to stain cell walls and naphtol blue black to stain proteins. Images were acquired using a stereoscopy MZFL III (Leica).

### DNA Methylation

Genomic DNA from *C. canephora* was extracted according to the protocol described by Echevarría-Machado et al. [70]. Briefly, 100 mg of explants under embryogenic induction conditions were collected every seven days from 0 to 56 days, and from somatic embryos at different developmental stages (proembryogenic mass, globular, heart, torpedo, cotyledonary and plantlets) and zygotic embryos. Nucleic acid digestion and the separation of the nucleosides is described in detail by De-la-Peña et al. [71]. Briefly, 5 µg of DNA from each sample were hydrolyzed and mixed with 5 µL of 10X DNA digestion buffer (200 mM acetic acid, 200 mM glycine, 50 mM magnesium chloride, 5 mM zinc acetate, 2 mM calcium chloride adjusted with sodium hydroxide to pH 5.3), 2 µL of DNase I (D2821-Sigma, 10 U/µL) and 1 µL of Nuclease P1 (N8630-Sigma, 1.25 U/µL). After overnight incubation at 37°C, the samples were mixed with 5 µL of 100 mM NaOH and 2 µL calf intestine alkaline phosphatase (P4879-Sigma, 1 U/µL). The samples were incubated for 3.5 h at 37°C and mixed with the mobile phase D (50 mM ammonium phosphate dibasic, 15 mM ammonium acetate adjusted with phosphoric acid to pH 4.1). After that, the samples were centrifuged at 18,000×g and analyzed by high performance liquid chromatography (HPLC, Agilent 1200 series). DNA methylation percentages were obtained from the phase-reversed chromatograms, using the peak areas to determine the concentration of 2′-deoxycytosine (dC) and 5-methyl-2′-deoxycytosine (5 mdC) in the sample (% 5 mdC = *C* 5 mdC/[*C* 5 mdC+*C* dC]×100), where *C* is concentration. All the analyses were performed with three biological replicates from different DNA extractions.

### 5-Azacytidine Assay

Embryogenic cultures of *C. canephora* were incubated in the absence (control) or presence of 10 and 20 µM of 5-azacytidine (Sigma) diluted in the same medium used for embryogenic induction. This compound was added into the medium every seven days starting at different independent time points (day 7, 14, 21 and 35) of the SE culture. Then the somatic embryos at each embryogenic stage in the control and the treatments with 5-AzaC were counted after 56 days’ induction. The percentage of DNA methylation under the effect of 5-AzaC was conducted as described above. Three independent assays were evaluated.

### Histone Isolation and Western Blots

Histones from *C. canephora* were isolated from 0.5 g of explants under embryogenic induction conditions and somatic embryos in different embryogenic stages (proembryogenic mass, globular, heart, torpedo and cotyledonary), as described by Nic-Can and De-la-Peña [72]. Briefly, ten micrograms of protein were separated by 15% SDS-gel page and blotted on PVDF membrane (Millipore Immobilon P) for 3.5 h at 265 mA. Membranes were blocked with 5% non-fat dry milk, 0.5% Tween in phosphate-buffered saline (TPBS). In all experiments, Millipore antibodies were used as follows: anti-H3 (cat. # 07-690) as a loading control, anti-dimethyl-histone H3 [Lys-4] (cat. # 07-030), anti-trimethyl-histone H3 [Lys-4] (cat. # 04-745), anti-dimethyl-histone H3 [Lys-9] (cat. # 07-441), anti-trimethyl-histone H3 [Lys-27] (cat. # 07-449). The primary antibodies were incubated at 4°C for either one hour or overnight with constant agitation. After washing three times for 10 min with 1X TPBS and one time with PBS, the membrane was incubated with secondary antibody Goat Anti-Rabbit IgG, HRP-conjugate (cat. # 12-348). The signal detection was achieved with the agent Immobilon Western HRP substrate peroxidase solution (Millipore) following the manufacturer’s instructions. Data from three independent analyses consistently gave the same results.

### Sequence Analysis and Primers Design

To design primers to amplify SE-related genes in *C. canephora,* the selection of nucleotide sequences was carried out, annotated by several databases listed in Table S1. Nucleotide alignments in multiple sequences were performed using the software ClustalW2-Multiple Sequence Alignment (http://www.ebi.ac.uk/) and a highly conserved region was chosen for the primers’ design. Primers for *LEC1, BBM1* and *WOX4* were realized with the software Primer 3 Plus (http://www.bioinformatics.nl/cgi-bin/primer3plus/primer3plus.cgi), and then were analyzed through the online programs Oligo Analyzer 3.1 (http://www.idtdna.com/analyzer/Applications/OligoAnalyzer/Default.aspx) and DNA calculator (http://www.sigma-genosys.com/calc/DNACalc.asp). Primers generated for RT-PCR are listed in Table S2.

### RT-PCR Analysis

Total RNA was extracted from 100 mg of leaf explants under embryogenic induction and were collected at 0, 7, 14 and 21 days, and from somatic embryos that were isolated and classified according to developmental stage (proembryogenic mass, globular, heart, torpedo and cotyledonary) and a zygotic embryo in the cotyledonary stage of *C. canephora* was used as a comparison. Tissue samples were homogenized with TRI reagent (Sigma) following the manufacturer’s instructions, and the quality of extracted RNA was verified on agarose gel at 1.5%. The quantity was verified in a Nanodrop (Thermo Fisher Scientific). For cDNA synthesis, reverse transcription reactions were performed in a 20-µL volume containing 1.5 µg of RNA and 200 U of the SuperScript™ II Reverse Transcriptase (Invitrogen) according to the manufacturer’s instructions. Platinum Taq polymerase (1.25 U, Invitrogen), 10 mM dNTPs, 10 µM each primer (listed in Table S2) in a 25-µL volume was used during PCR and the conditions were listed as follows: for *BBM1,* 95°C for 4 min, followed by 35 cycles of 95°C for 40 sec, 65°C for 45 sec, 72°C for 90 sec and a final cycle of 72°C for 10 min; for *LEC1, WOX4* and *UBQ11*, 95°C for 5 min, followed by 30 cycles of 95°C for 40 sec, 60°C for 45 sec, 72°C for 70 sec and a final cycle of 72°C for 5 min. The PCR products were electrophoresed in a 1.5% agarose gel and stained with GelRed (Biotium), and the images were acquired. Band intensities were quantified using the Gel Doc™ XR+System (BIO-RAD) and the intensities of genes mentioned above were normalized to the constitutive gene *UBQ11*. Each RT-PCR was conducted twice with three biological replicates.

### Chromatin Immunoprecipitation (ChIP) Analysis

Explants under embryogenic conditions and somatic embryos (0 and 14 days, proembryogenic mass, heart, torpedo and cotyledonary) were vacuum-infiltrated with formaldehyde crosslinking solution. ChIP experiments were performed as described previously by De-la-Peña el at. [73], with slight modifications. In two biological replicates, the chromatin was immunoprecipitated using the following antibodies obtained from Millipore: anti-trimethyl-histone H3 [Lys-4] (cat. # 04-745), anti-dimethyl-histone H3 [Lys-9] (cat. # 07-441), anti-trimethyl-histone H3 [Lys-27] (cat. # 07-449) and anti-dimethyl-histone H3 [Lys-36] (cat. # 07-274). PCR amplifications were done in 25 µL volumes using the following conditions: for *LEC1, WOX4* and *UBQ11*, 95°C for 5 min, followed by 40 cycles of 95°C for 40 sec, 60°C for 50 sec, 72°C for 2 min and a final cycle of 72°C for 10 min and for *BBM1,* 95°C for 5 min, followed by 40 cycles of 95°C for 40 sec, 65°C for 50 sec, 72°C for 2 min and a final cycle of 72°C for 10 min. Primer sequences are shown in Table S3. The PCR products were electrophoresed in a 1.5% agarose gel and stained with GelRed (Biotium) and the images were acquired using the Gel Doc™ XR+System (BIO-RAD). *UBQ* is a constitutively active gene that carries both trimethylated Lys 4 and dimethylated Lys 9 of histone H3 and has been used as a control [71], [74].

### Statistical Analysis

All the data were processed and analyzed using an analysis of variance (ANOVA). The significance level between the mean values was carried out using the Tukey test. Differences were considered to be significant at *P≤*0.05. Data were analyzed by Origin 8 (Data Analysis and Graphing Software).

### Comparative Bioinformatic Analysis

To evaluate the ChIP results generated in this study, the epigenomics data (histone modifications and DNA methylation) from *Arabidopsis* and rice were analyzed through the publicly accessible database of Vincent Colot for *Arabidopsis* (http://epigara.biologie.ens.fr/index.html) and the RMAP-A Map Like Rice Genome Browser [75] (http://www.ricemap.org/gmap.jsp) using the orthologous genes as follows: AT1G21970 (*LEC1*), AT5G17430 (*BBM1*), AT1G46480 (*WOX4*), LOC_Os02g49370 (*LEC1*), LOC_Os04g42570 (*BBM1*), and LOC_Os04G55590 (*WOX4*).

## Supporting Information

Figure S1 **Global DNA methylation analysis of leaf explants during somatic embryogenic induction exposed to 5-Azacytidine (5-AzaC).** Leaf explants of *Coffea canephora* were treated with 10 µM 5-AzaC every 7 days, from day 7 until 56 days, as shown in Figure 5A (7 dai), and DNA methylation levels were measured by HPLC as described in Materials and Methods. Error bars represent ± SE (n = 3). The experiment was carried out three times.
(TIF)

Figure S2 **Amino acid sequence alignment of the B domains of plant LEC1 proteins.** Identical residues are marked with stars. The DNA-binding region and subunit interaction are highlighted in yellow boxes. The position of α-helices and loops in the histone fold motif is indicated with black and red lines, respectively. The Asp (D) residue that is required for the *LEC1* function is shaded in orange. The consensus sequence that interacts with the TATA-binding protein is highlighted in red. *Zm, Zea mays* (*Zm*LEC1); *Os, Oryza sativa* (*Os*LEC1); *Tc, Theobroma cacao* (*Tc*LEC1-Like); *Dc, Daucus carota* (*Dc*LEC1); *Cc*, *Coffea canephora* (*Cc_*GT656663.1); *Is, Isoetes sinensis* (*Is*CAAt-Box); *At, Arabidopsis thaliana* (*At*LEC1); *Bn, Brassica napus* (*Bn*LEC1); *Pc, Pistacia chinensis* (*Pc*LEC1); *Mt, Medicago truncatula* (*Mt*LEC1).
(TIF)

Figure S3 **Amino acid sequence alignment of plants’ BBM1 proteins.** Identical residues are marked with stars. Amino acid sequences of the first AP2/ERF domain repeat (Repeat 1) and the second AP2/ERF domain repeat (Repeat 2) are highlighted in blue and the linker region that joins the two repeats is highlighted in yellow. Red boxes indicate the overlap of both sequences of *Coffea canephora*: *Cc*GT656297.1 and *Cc*GT656313.1. *At, Arabidopsis thaliana* (*At*BBM1); *Bn, Brassica napus* (*Bn*BBM1); *Gm, Glycine max* (*Gm*BBM1); *Mt, Medicago truncatula* (*Mt*BBM1); *Tc, Theobroma cacao* (*Tc*BBM1); *Vv, Vitis vinifera* (*Vv*BBM1); *Zm, Zea mays* (*Zm*BBM1); *Os, Oryza sativa* (*Os*BBM1).
(TIF)

Figure S4 **Amino acid sequence alignment of WOX4.** The homeodomain that binds DNA through a helix (gray boxes) turn helix structure of WOX4 is shown. The homeodomain proteins used in the alignment were as follows: *Sl, Solanum lycopersicum* (*Sl*WOX4); *Vv, Vitis vinifera* (*Vv*WOX4); *Cc*, *Coffea canephora* (SNG U627534); *Gm, Glycine max* (*Gm*WOX4); *At, Arabidopsis thaliana* (*At*WOX4); *Pt, Populus trichocarpa* (*Pt*WOX4); *Os, Oryza sativa* (*Os*WOX4); *Bd, Brachypodium distachyon* (*Bd*WOX4); *Zm, Zea mays* (*Zm*WOX4).
(TIF)

Figure S5 **Genome browser view of epigenetic modifications of** ***LEC1*** **,** ***BBM1*** **and** ***WOX4*** **in** ***Arabidopsis thaliana.*** **A)** Representative genes model of *LEC1*, *BBM1* and *WOX4* sequences. The green boxes show the exons, the connecting lines are the introns and the red boxes are the untranslated regions (UTRs). The black line below the genes represents the analyzed region in *Coffea canephora*. Epigenetic modifications in a genomic region of **B)** *LEC1* (AT1G21970), **C)** *BBM1* (AT5G17430) and **D)** *WOX4* (AT1G46480). A select region indicated by the red dashed lines represents the compared position vs. ChIP of *LEC1*, *BBM1* and *WOX4* in the somatic embryogenesis of *C. canephora.*
(TIF)

Figure S6 **Genome browser view of epigenetic modifications of** ***LEC1*** **,** ***BBM1*** **and** ***WOX4*** **in** ***Oriza sativa.*** **A)** Representative genes model of *LEC1*, *BBM1* and *WOX4* sequences. The green boxes show the exons, the connecting lines are the introns and the red boxes are the untranslated regions (UTRs). The black line below represents the region analyzed in *Coffea canephora*. Epigenetic modifications in a genomic region of **B)** *LEC1* (LOC_Os02g49370), **C)** *BBM1* (LOC_Os04g42570) and **D)** *WOX4* (LOC_Os04G55590). A select region indicated by the red dashed lines represents the compared position vs. ChIP of *LEC1*, *BBM1* and *WOX4* in the somatic embryogenesis of *C. canephora.*
(TIFF)

Table S1 Genes used in motif analysis.
(DOCX)

Table S2 Primers used in RT-PCR experiments.
(DOCX)

Table S3 Primers used to Chromatin Immunoprecipitation (ChIP) assays.
(DOCX)
