# Supplementary figures and images for: New Insights into Somatic Embryogenesis: LEAFY COTYLEDON1, BABY BOOM1 and WUSCHEL-RELATED HOMEOBOX4 Are Epigenetically Regulated in Coffea canephora
Source: PLoS One. 2013 Aug 20;8(8):e72160. doi: 10.1371/journal.pone.0072160 (PMC3748027; doi:10.1371/journal.pone.0072160)

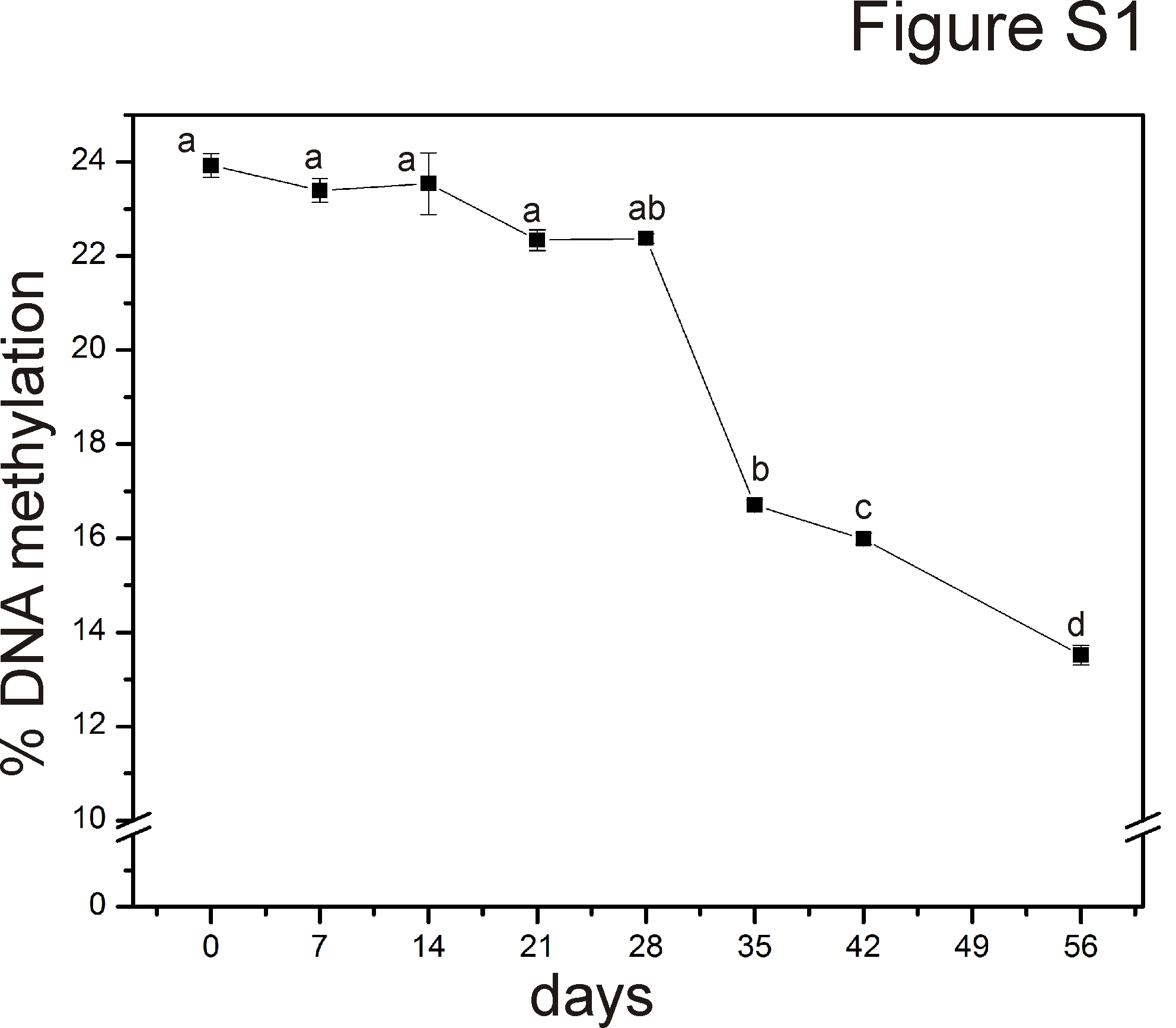

Supplement: Figure S1 — Global DNA methylation analysis of leaf explants during somatic embryogenic induction exposed to 5-Azacytidine (5-AzaC). Leaf explants of Coffea canephora were treated with 10 µM 5-AzaC every 7 days, from day 7 until 56 days, as shown in Figure 5A (7 dai), and DNA methylation levels were measured by HPLC as described in Materials and Methods. Error bars represent ± SE (n = 3). The experiment was carried out three times. (TIF) [file pone.0072160.s001.tif]

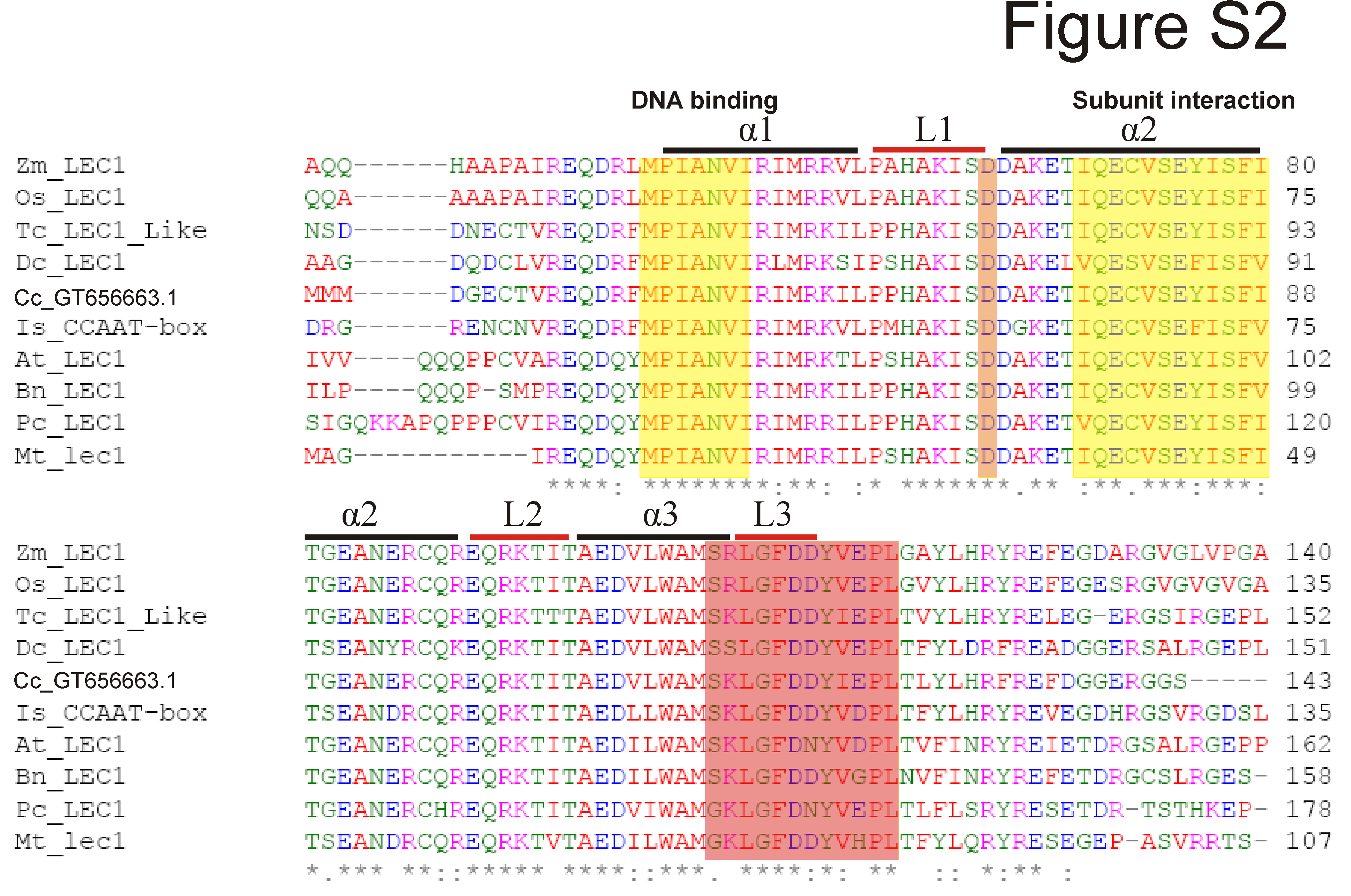

Supplement: Figure S2 — Amino acid sequence alignment of the B domains of plant LEC1 proteins. Identical residues are marked with stars. The DNA-binding region and subunit interaction are highlighted in yellow boxes. The position of α-helices and loops in the histone fold motif is indicated with black and red lines, respectively. The Asp (D) residue that is required for the LEC1 function is shaded in orange. The consensus sequence that interacts with the TATA-binding protein is highlighted in red. Zm, Zea mays (ZmLEC1); Os, Oryza sativa (OsLEC1); Tc, Theobroma cacao (TcLEC1-Like); Dc, Daucus carota (DcLEC1); Cc, Coffea canephora (Cc_GT656663.1); Is, Isoetes sinensis (IsCAAt-Box); At, Arabidopsis thaliana (AtLEC1); Bn, Brassica napus (BnLEC1); Pc, Pistacia chinensis (PcLEC1); Mt, Medicago truncatula (MtLEC1). (TIF) [file pone.0072160.s002.tif]

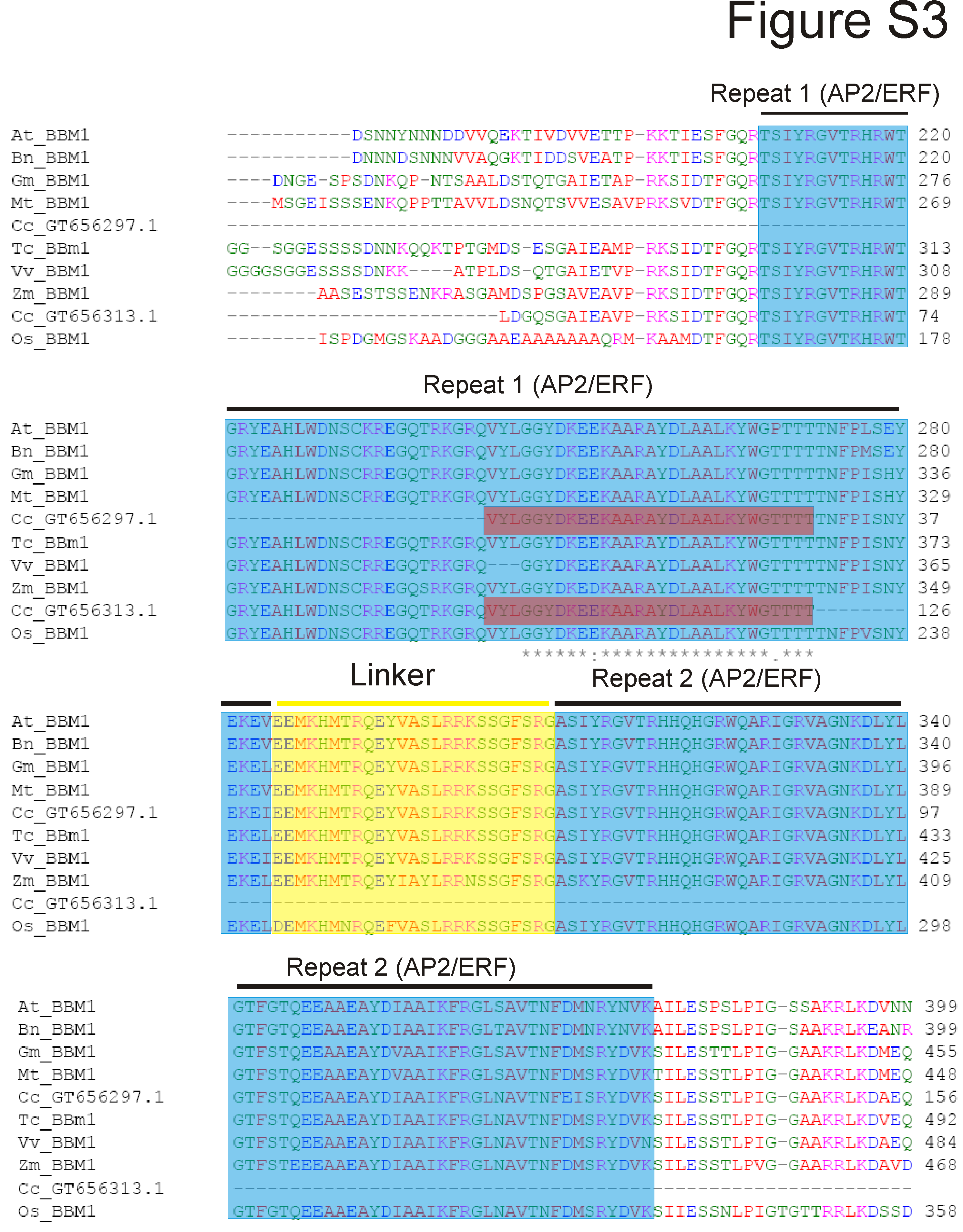

Supplement: Figure S3 — Amino acid sequence alignment of plants’ BBM1 proteins. Identical residues are marked with stars. Amino acid sequences of the first AP2/ERF domain repeat (Repeat 1) and the second AP2/ERF domain repeat (Repeat 2) are highlighted in blue and the linker region that joins the two repeats is highlighted in yellow. Red boxes indicate the overlap of both sequences of Coffea canephora: CcGT656297.1 and CcGT656313.1. At, Arabidopsis thaliana (AtBBM1); Bn, Brassica napus (BnBBM1); Gm, Glycine max (GmBBM1); Mt, Medicago truncatula (MtBBM1); Tc, Theobroma cacao (TcBBM1); Vv, Vitis vinifera (VvBBM1); Zm, Zea mays (ZmBBM1); Os, Oryza sativa (OsBBM1). (TIF) [file pone.0072160.s003.tif]

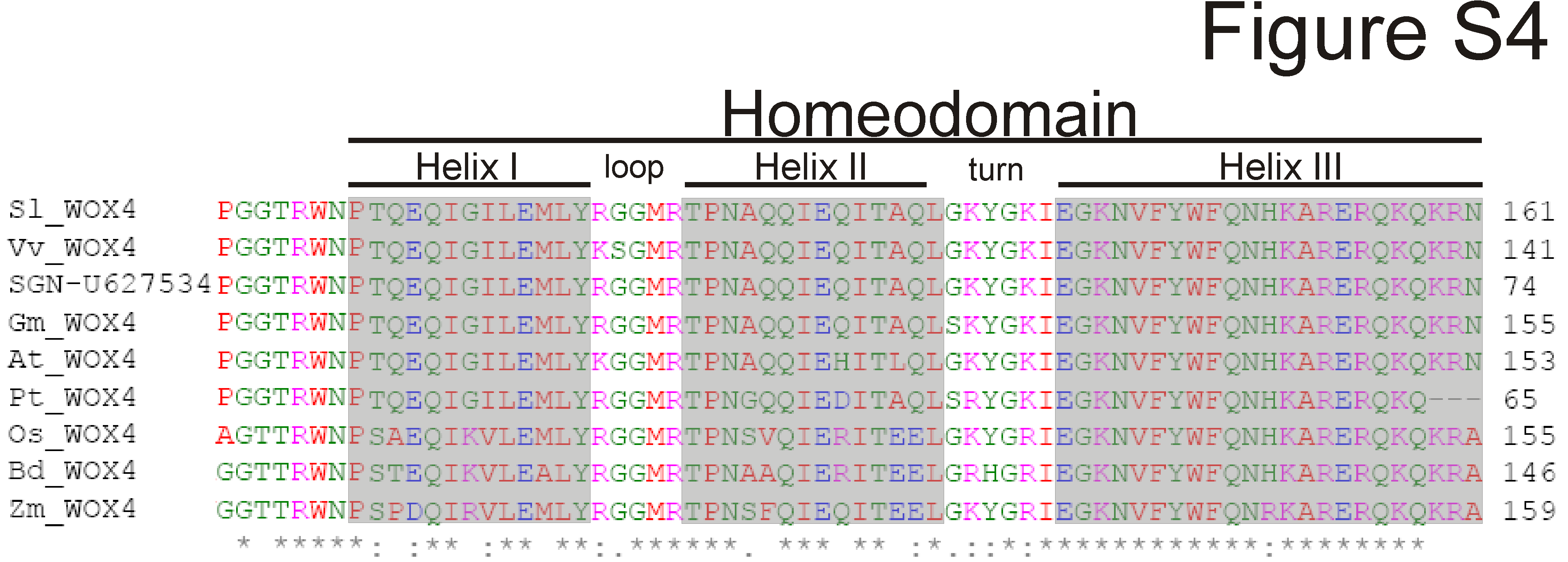

Supplement: Figure S4 — Amino acid sequence alignment of WOX4. The homeodomain that binds DNA through a helix (gray boxes) turn helix structure of WOX4 is shown. The homeodomain proteins used in the alignment were as follows: Sl, Solanum lycopersicum (SlWOX4); Vv, Vitis vinifera (VvWOX4); Cc, Coffea canephora (SNG U627534); Gm, Glycine max (GmWOX4); At, Arabidopsis thaliana (AtWOX4); Pt, Populus trichocarpa (PtWOX4); Os, Oryza sativa (OsWOX4); Bd, Brachypodium distachyon (BdWOX4); Zm, Zea mays (ZmWOX4). (TIF) [file pone.0072160.s004.tif]

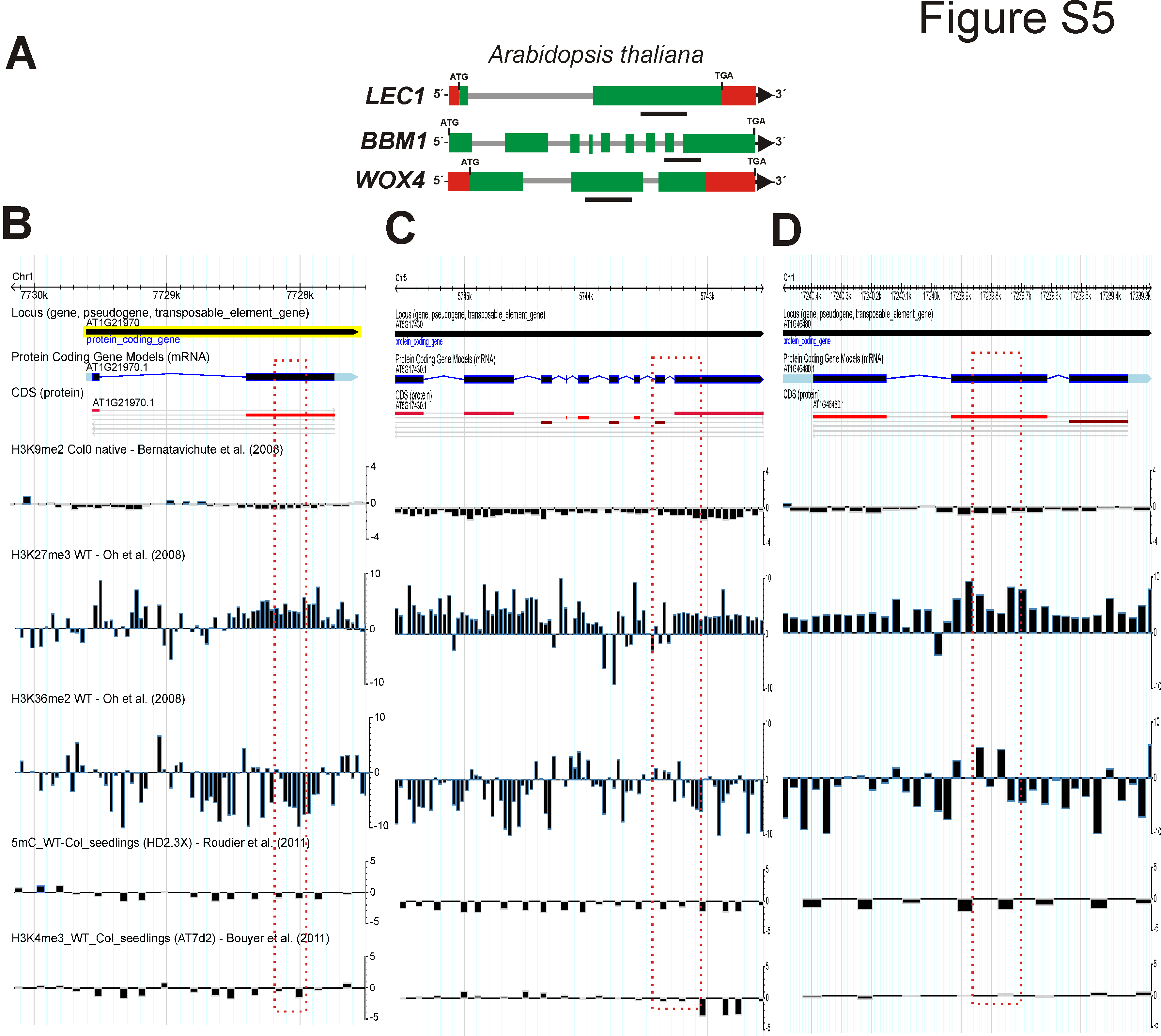

Supplement: Figure S5 — Genome browser view of epigenetic modifications of LEC1 , BBM1 and WOX4 in Arabidopsis thaliana. A) Representative genes model of LEC1, BBM1 and WOX4 sequences. The green boxes show the exons, the connecting lines are the introns and the red boxes are the untranslated regions (UTRs). The black line below the genes represents the analyzed region in Coffea canephora. Epigenetic modifications in a genomic region of B) LEC1 (AT1G21970), C) BBM1 (AT5G17430) and D) WOX4 (AT1G46480). A select region indicated by the red dashed lines represents the compared position vs. ChIP of LEC1, BBM1 and WOX4 in the somatic embryogenesis of C. canephora. (TIF) [file pone.0072160.s005.tif]

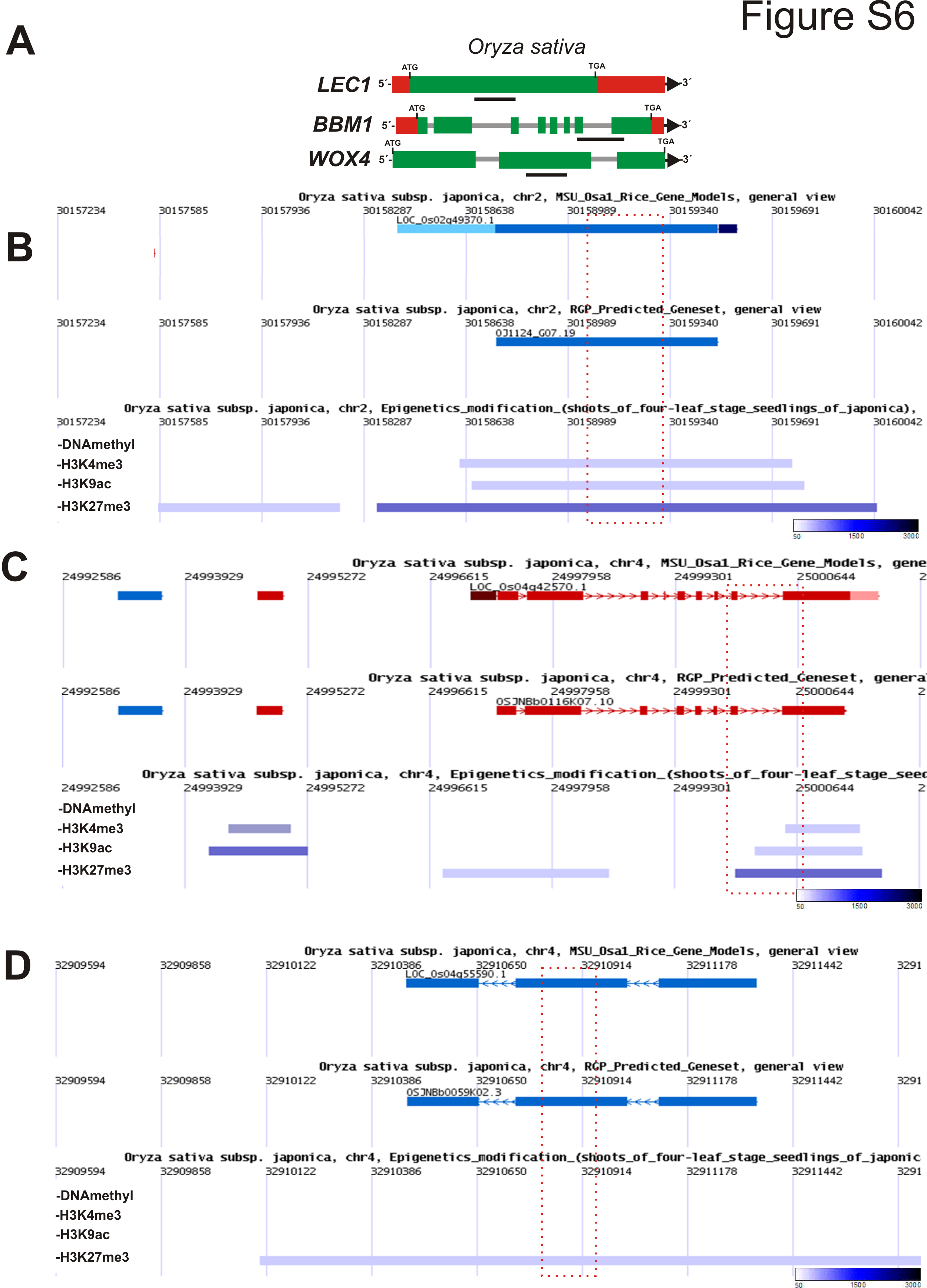

Supplement: Figure S6 — Genome browser view of epigenetic modifications of LEC1 , BBM1 and WOX4 in Oriza sativa. A) Representative genes model of LEC1, BBM1 and WOX4 sequences. The green boxes show the exons, the connecting lines are the introns and the red boxes are the untranslated regions (UTRs). The black line below represents the region analyzed in Coffea canephora. Epigenetic modifications in a genomic region of B) LEC1 (LOC_Os02g49370), C) BBM1 (LOC_Os04g42570) and D) WOX4 (LOC_Os04G55590). A select region indicated by the red dashed lines represents the compared position vs. ChIP of LEC1, BBM1 and WOX4 in the somatic embryogenesis of C. canephora. (TIFF) [file pone.0072160.s006.tif]
