# Supplementary material for: New Insights into Somatic Embryogenesis: LEAFY COTYLEDON1, BABY BOOM1 and WUSCHEL-RELATED HOMEOBOX4 Are Epigenetically Regulated in Coffea canephora
Source: PLoS One. 2013 Aug 20;8(8):e72160. doi: 10.1371/journal.pone.0072160 (PMC3748027; doi:10.1371/journal.pone.0072160)
Supplement: Table S1 — Genes used in motif analysis. (DOCX) [file pone.0072160.s007.docx]

| **Gene** | **Accession number** |  | **Data base** |
| --- | --- | --- | --- |
| ***LEC1*** | AT1G21970 | *Arabidopsis thaliana* | TAIR |
|  | EU371726.1 | *Brassica napus* | GenBank |
|  | AM494833.1 | *Theobroma cacao* | GenBank |
|  | HM627254.1 | *Pistacia chinensis* | GenBank |
|  | EF108293.1 | *Isoetes sinensis* | GenBank |
|  | Medtr1g039040.1 | *Medicago truncatula* | JCVI |
|  | AB104611.1 | *Daucus carota* | GenBank |
|  | AY264284.1 | *Oryza sativa* | GenBank |
|  | AF410176.1 | *Zea mays* | GenBank |
|  | ^a^GT656663.1 | *Coffea canephora* | GenBank |
|  |  |  |  |
| ***BBM1*** | AT5G17430 | *Arabidopsis thaliana* | TAIR |
|  | AF317904.1 | *Brassica napus* | GenBank |
|  | CGD0023756 | *Theobroma cacao* | SHRS cacao DB |
|  | HM775856.1 | *Glycine max* | GenBank |
|  | AY899909.1 | *Medicago truncatula* | GenBank |
|  | XM_002269804.2 | *Vitis vinifera* | GenBank |
|  | Os11g19060.1 | *Oryza sativa* | TIGR |
|  | EU955732.1 | *Zea mays* | GenBank |
|  | ^a^GT656313.1 | *Coffea canephora* | GenBank |
|  | ^a^GT656297.1 | *Coffea canephora* | GenBank |
|  |  |  |  |
| ***WOX4*** | ^a^U627534 | *Coffea canephora* | SNG |
|  | NP_001234251.1 | *Solanum lycopersicum* | GenBank |
|  | XP_002284927.1 | *Vitis vinifera* | GenBank |
|  | XP_003544467.1 | *Glycine max* | GenBank |
|  | NP_175145.2 | *Arabidopsis thaliana* | GenBank |
|  | CAJ84151.1 | *Populus trichocarpa* | GenBank |
|  | NP_001054082.1 | *Oryza sativa* | GenBank |
|  | CAM32347.1 | *Zea mays* | GenBank |
|  | XP_003579392.1 | *Brachypodium distachyon* | GenBank |

**Table S1.**  Genes used in motif analysis.

Genes of several species were retrieved from different databases and the accession numbers are also indicated. Genbank- genetic sequence database; TAIR –The Arabidopsis Information Resource; JCVI - J. Craig Venter Institute *Medicago truncatula* database; SHRS cacao DB - cacao genome database; TIGR - TIGR Rice Genome Annotation Project Database; EMBL-EBI - European Bioinformatics Institute; SNG-Sol genomics network. ^a^ sequences of *Coffea Canephora* related to gene under study.
