# Supplementary material for: New Insights into Somatic Embryogenesis: LEAFY COTYLEDON1, BABY BOOM1 and WUSCHEL-RELATED HOMEOBOX4 Are Epigenetically Regulated in Coffea canephora
Source: PLoS One. 2013 Aug 20;8(8):e72160. doi: 10.1371/journal.pone.0072160 (PMC3748027; doi:10.1371/journal.pone.0072160)
Supplement: Table S3 — Primers used to Chromatin Immunoprecipitation (ChIP) assays. (DOCX) [file pone.0072160.s009.docx]

**Table S3**. Primers used to Chromatin Immunoprecipitation (ChIP) assays.

| **Gene** | **Primers Forward and Reverse** | **Annealing T (°C)** | | **Size of product (bp)** |
| --- | --- | --- | --- | --- |
| ***LEC1*** | F-5´- ATGATGAGAGCAGCAGAGATAAGC -3´  R-5´- GGGTTAGAGGCTCAATGTAGTCAT -3´ | | 60 | 379 |
| ***BBM1*** | F-5´- TACGTTGCATCTTTACGAAGGAAG -3´  R-5´- TATGAGCATCCAAAGCCATTTCAG 3´ | | 65 | 340 |
| ***WOX4*** | F-5´- GGAGGGACGAGGTGGAATCCA-3´  R-5´- CTACTAATGGTAGTGGTGGGGTGA-3´ | | 60 | 262 |
| ***UBQ11*** | F-5´-GACGGGCGCACCCTTGCGGATTAC-3´  R-5'-TCCTGGATCTTCGCCTTGACATTG-3´ | | 60 | 211 |
